# Preliminary clinical assessment of the relationship between tumor alphavbeta3 integrin and perfusion in patients studied with [^18^F]fluciclatide kinetics and [^15^O]H_2_O PET

**DOI:** 10.1186/s13550-014-0030-x

**Published:** 2014-08-08

**Authors:** Laura M Kenny, Giampaolo Tomasi, Federico Turkheimer, James Larkin, Martin Gore, Cathryn S Brock, Stephen Mangar, Eric O Aboagye

**Affiliations:** Department of Surgery and Cancer, Hammersmith Campus, Imperial College London, London, UK; Department of Medical Oncology, Imperial College Healthcare NHS Trust, London, UK; Centre for Neuroimaging, Institute of Psychiatry, King’s College London, London UK; Royal Marsden NHS Trust, London, UK

**Keywords:** Fluciclatide, Perfusion, Angiogenesis, PET, Cancer

## Abstract

**Background:**

[^18^F]fluciclatide, a peptide ligand with high affinity for αvβ3/αvβ5 integrins, is a proposed biomarker of tumor angiogenesis. The study rationale was to perform a preliminary evaluation of the relationship between tumor [^18^F]fluciclatide uptake and perfusion by [^15^O]H_2_O PET.

**Methods:**

Patients with non-small cell lung cancer and melanoma underwent dynamic imaging with arterial sampling following injection of [^15^O]H_2_O and [^18^F]fluciclatide. Quantification was performed using a one-tissue compartmental model for [^15^O]H_2_O and a two-tissue model for [^18^F]fluciclatide at volume-of-interest level, and SUV at voxel level.

**Results:**

Tumor binding potential (*k*_3_/*k*_4_ ratio) of [^18^F]fluciclatide tumor was 5.39 ± 1.46, consistent with previous studies in breast cancer metastases. Voxel-by-voxel maps of [^18^F]fluciclatide delivery strongly correlated with [^15^O]H_2_O-based perfusion (*p* < 10^−4^ tumor, 1,794 ± 1,331 voxels). Interestingly, this correlation was lost when retention of [^18^F]fluciclatide at late time-points was compared with perfusion (*p* > 0.15).

**Conclusions:**

Our study suggests tumor [^18^F]fluciclatide retention is unrelated to tumor perfusion, supporting use of late (60-min) imaging protocols in patients.

**Electronic supplementary material:**

The online version of this article (doi:10.1186/s13550-014-0030-x) contains supplementary material, which is available to authorized users.

## Background

Tumor angiogenesis is essential for cancer survival [1]. Many novel therapeutics have been developed which aim to target this process by acting as either angiogenesis inhibitors (e.g. by blocking the action of vascular endothelial growth factor) or as vascular disrupting agents, which target the extracellular matrix [2],[3]. Despite the recent advances in therapeutics, there remains a paucity of effective biomarkers which can predict the response of tumors *in vivo* to these treatments. One area of promise is the development of imaging agents that target the αvβ3 and αvβ5 integrins - a set of transmembrane proteins essential for maintaining the relationship between the cell and the extracellular matrix and that have been found to be upregulated on tumor vascular neoendothelium [4],[5].

[^18^F]fluciclatide is an arginine-glycine-aspartic acid (RGD) peptide which binds the αvβ3 and αvβ5 integrins with high affinity [6]. We have previously studied the dosimetry and biodistribution of this compound labelled with F-18 in healthy volunteers with positron emission tomography (PET) [7] and the uptake profile of the tracer in patients with metastatic breast cancer [8]. The latter study showed significantly higher radiotracer uptake in lung metastases from breast cancer compared to normal lung tissue. [^18^F]galacto-RGD is a similar promising agent for studying integrin-related angiogenesis developed by Wester's group [9], and subsequently studied by Beer et al. who demonstrated that there is high uptake of this compound in a variety of cancers including melanoma [10] and that uptake correlates with expression of the αvβ3 integrin measured using immunohistochemistry [11].

The purpose of this study was to understand the relationship between perfusion assessed by [^15^O]H_2_O and ^18^F-fluciclatide kinetics in non-small cell lung cancer (NSCLC) and melanoma, where Phase 1 trials of humanized antibodies and peptides targeted to integrins have shown promise [12],[13]. These two tumor types have also been shown to have varying degrees of αvβ3 and αvβ5 expression on tumor cells relative to vascular endothelium with melanoma suggested to have expression on both tumor and vascular endothelium and NSCLC suggested to have expression predominantly on the vascular endothelium [4]. The underlying hypothesis for the study was that fluciclatide retention in tumors is initially dependent on perfursion, but at later timepoints, retention is independent of perfusion.

## Methods

Patients were recruited from oncology clinics at the Imperial College NHS Healthcare trust and the Royal Marsden Hospital NHS Trust. The study was approved by the hospital Local Research Ethics Committee in accordance with the Helsinki Declaration revised guidelines (2008). The eligibility criteria were as follows: patients aged ≤80 years old with a histological proven NSCLC or melanoma, and one site of measureable disease of ≥2-cm diameter, with a treatment-free interval of 3 weeks (or 4 weeks for radiotherapy to the imaging site), life expectancy of at least 3 months, haemoglobin ≤10 g/dL, granulocyte count of ≥1.5 × 10^9^, platelet count of ≥100 × 10^9^, adequate hepatic function; exclusion criteria: pregnant or lactating patients, sexually active patients who are not employing adequate contraception. Additional details are provided in Table 1.

**Table 1:** **Demographics of the patients studied**

Patient	Age	Diagnosis	Stage	Dynamic imaging region
1	81	Melanoma	IV	Thorax
2	62	NSCLC*	IIIA	Thorax
3	57	NSCLC	IIIB	Thorax/Neck
4	59	NSCLC	IB	Thorax
5	66	NSCLC	IV	Thorax
6	79	Melanoma	IV	Knee
7	59	NSCLC	IV	Thorax

NSCLC, non-small cell lung cancer.

### Radiosynthesis of ^18^F-fluciclactide and PET scanning procedure

The chemical synthesis of the precursor of fluciclatide has previously been described [6], radiolabelling was performed at Hammersmith Imanet as previously described on an automated module (TRACERlab FX _F-N_; GE Healthcare). The specific activity and radiochemical purity of the injectate, determined by high-performance liquid chromatography (HPLC), was 320 GBq/μmol and 99.7 %, respectively.

All patients were scanned on a PET-CT (GE-Discovery RX) scanner after being positioned such that the field of view (FOV) included the tumor volume of interest. The axial FOV of the scanner was 15.7 cm and the transaxial FOV was 70 cm. A low-dose CT scan (20 mA and 120 kV) was used for attenuation correction prior to the PET scan. A target dose of 600 MBq [^15^O]H_2_O was injected i.v. over 20 s followed by a 30-s flush with normal saline. Dynamic PET scanning was done for 8 min and 40 s with the following frame durations: 30 s × 1, 20 s × 1, 5 s × 22, 10 s × 3, 30 s × 5, and 60 s × 3. After a 2-min break to allow for radioactive decay (^15^O half-life = 2.04 min), the [^18^F]fluciclatide scan was performed. [^18^F]fluciclatide was administered by a bolus i.v. injection over 10 to 30 s. The PET acquisition was conducted as a single bed position dynamic scan (three-dimensional acquisition) centred on the tumor volume of interest (VOI) for 66.5 min. Data were binned into the following time frames: 10 s × 10, 20 s × 4, 30 s × 4, 60 s × 7, 120 s × 4, 300 s × 3, and 600 s × 3. Sinograms were Fourier-rebinned into two-dimensional slices and reconstructed (with correction for attenuation, scatter, and dead time) using filtered back-projection (ramp filter kernel full-width-at-half-maximum of 2.0 mm). The final images had 128 × 128 × 47 voxels of 2.62 × 2.62 × 2.42 mm^3^.

In order to derive an input function, blood samples were taken during the [^15^O]H_2_O scan *via* a radial artery cannula continuously at a rate of 5 mL/min for the first 9 min 40 s; discrete samples were taken at 3, 6, and 8 min 40 s. Similarly, continuous arterial sampling (5 mL/min) was performed for 10 min during the [^18^F]fluciclatide scan; seven discrete samples (5 to 10 mL) were taken at 2.5, 5, 10, 15, 30, 45, 60 min. Metabolite analyses were performed on the 2.5-, 5-, 10-, 30-, and 60-min samples. The computation of [^18^F]fluciclatide parent fraction and input function was carried out as described in [7].

### Data analysis

Volumes-of-interest (VOIs) were manually defined around visible tumors from the [^18^F]fluciclatide summed images using the Analyze software (Version 7). The same VOIs were applied to the [^15^O]H_2_O scan to extract tumor time-activity curves (TACs) for both tracers.

To estimate the perfusion from the [^15^O]H_2_O data, a one-tissue compartment model was employed:

1 $$C_{T}\left(t\right)=\left(1-V_{b}\right)\times \left[K_{1}\times \left[C_{a}\left(t\right)⊗\exp\left(-k_{2}\times t\right)\right.\right]+V_{b}C_{a}\left(t\right)$$

In Equation 1, *C*_T_(*t*) indicates the tumor TAC, *C*_a_(*t*) the measured radioactivity concentration in arterial blood*, K*_1_ the regional blood flow (mL/cm^3^/min), *k* 2 (1/min) the transfer rate from tissue to blood, *V*_b_ (unitless) the blood volume fraction and ⊗ the convolution operator. *K*_1_, *k*_2_, and *V*_b_ were estimated for each VOI by fitting *C*_T_(*t*) to Equation 1 using the standard weighted non-linear least squares (WNLLS). WNLLS minimizes the weighted residual sum of squares (WRSS)

2 $$\mathrm{WRSS}\left(p\right)=\sum_{i=1}^{n}w_{i}{\left[C{\left(t_{i},p\right)}^{\mathrm{MODEL}}-C_{T}\left(t_{i}\right)\right]}^{2}$$

with *p*, *t*_*i*_, and *n* indicating respectively the parameter vector [*K*_1_;*k*_*2*_;*V*_b_], the mid-time of the *i* th frame, and the number of frames. In Equation 2 weights *wi* were set to

3 $$w_{i}=\frac{\Delta_{i}}{C\left(t_{i}\right)\exp\left(\lambda t_{i}\right)}$$

Tomasi G 2009, [14] with Δ_*i*_ and *λ* indicating respectively the duration of the *i* th frame and the half-life of ^15^O. The delay between *C*_a_(*t*) and *C*_T_(*t*) was modelled by shifting *C*_T_(*t*) of *i* seconds (*i* = 0,1, …, 30) and then retaining the value of the delay which gave rise to the smallest WRSS.

A two-tissue reversible compartment model was used to model [^18^F]fluciclatide kinetics, consistent with our previous study [8]. The metabolite-corrected measured arterial concentration of [^18^F]fluciclatide was used as input function for estimating with WNNLS the parameters *K*_1_ (mL/cm^3^/min), *k*_2_ (1/min), *k*_3_ (1/min), *k*_4_ (1/min), *V*_b_ (unitless) for each VOI. Weights were computed from Equation 3. The parameter *k*_3_/*k*_4_ ratio - a measure of the binding potential - was then computed for each VOI; as we previously reported [8], this parameter was the best of a number of parameters examined to differentiate between healthy and tumor VOIs.

*K*_1_, *k*_2,_ and *V*_b_ were also estimated at the voxel level for [^15^O]H_2_O using the standard Basis Function Method approach [15]. The final maps, however, were extremely noisy because of the high noise of [^15^O]H_2_O data and we did not include them in the analysis. Parametric maps were not generated in the case of [^18^F]fluciclatide because of the difficulty of fitting a five-parameter model at the voxel level. To analyze the correlation between perfusion and [^18^F]fluciclatide uptake at the voxel level we employed the following semi-quantitative parameters. For [^15^O]H_2_O we used

4 $$\mathrm{SU}V_{\mathrm{water}}\left[m^{2}/L\right]=\frac{\mathrm{mean}\left[C_{T}{\left(t\right)}_{0-1.5\min}\right]}{\frac{\mathrm{Injected}\;\mathrm{dose}}{\mathrm{BSA}}}$$

In Equation 4, we arbitrarily chose 1.5 min as the final time, instead of the scan duration of 8 min, to define a parameter describing [^15^O]H_2_O delivery (i.e. flow) before washout took place. To measure [^18^F]fluciclatide retention, we employed

5 $$\mathrm{SU}V_{\mathrm{fluci}\;\mathrm{RETENTION}}\;\left[m^{2}/L\right]=\frac{C_{T}\left(t_{i}\right)}{\frac{\mathrm{Injected}\;\mathrm{dose}}{\mathrm{BSA}}}$$

In Equations 4 and 5, BSA denotes the body surface area, and *t*_*i*_ in Equation 5 indicates the mid-time of the last frame of the [^18^F]fluciclatide scan (60.5 min). We also defined a parameter describing [^18^F]fluciclatide delivery as

6 $$\mathrm{SU}V_{\mathrm{fluci}\;\mathrm{DELIVERY}}\;\left[m^{2}/L\right]=\frac{\mathrm{mean}\;\left[C_{T}{\left(t\right)}_{0-1.5\min}\right]}{\frac{\mathrm{Injected}\;\mathrm{dose}}{\mathrm{BSA}}}$$

where the same upper limit used for SUVwater was employed. These parameters were computed for each voxel of the tumor VOIs and the agreement of SUVwater with SUVfluci RETENTION and SUVfluci DELIVERY was assessed using Spearman's correlation coefficient.

## Results

The activity of injected [^18^F]fluciclatide ranged between 290 and 383 MBq, and that of [^15^O]H_2_O between 448 and 697 MBq. Due to tracer failure and patient comfort reasons, PET scans of both tracers were carried out in four patients; for two patients, only the [^18^F]fluciclatide data were obtained, and for the remaining patient, only the [^15^O]H_2_O data were obtained.

**Figure 1:**
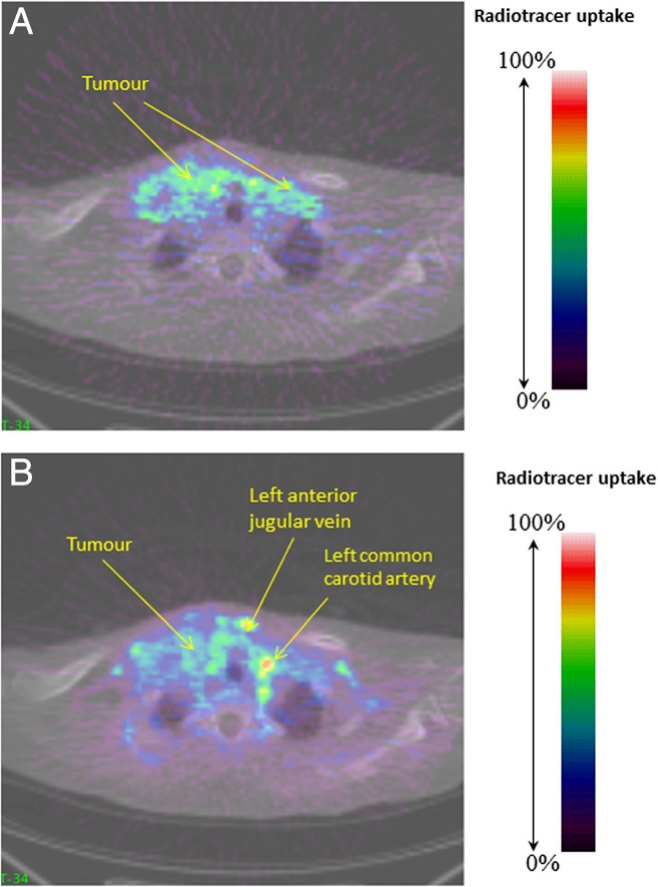
**Summed images of (A) [**^**15**^**O]H**_**2**_**O and (B) [**^**18**^**F]fluciclatide.** These were in a patient with non-small cell lung cancer with bilateral cervical lymph node metastases. The blood vessels are labelled.

**Figure 2:**
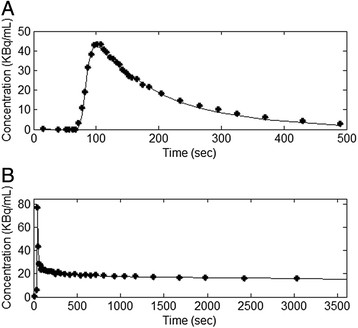
**Time-activity curves for (A) [** ^**15**^ **O]H**_**2**_ **O and (B) [** ^**18**^ **F]fluciclatide.**

Typical images of [^15^O]H_2_O and [^18^F]fluciclatide are shown in Figure 1A,B, respectively. The quality of the fits was good for both tracers. Examples are displayed in Figure 2A for [^15^O]H_2_O and Figure 2B for [^18^F]fluciclatide. In Table 2, the kinetic parameters for [^15^O]H_2_O and the *k*_3_/k_4_ ratios of [^18^F]fluciclatide at the VOI level are reported. For completeness, we also reported SUV parameters for [^18^F]fluciclatide.

**Table 2:** **Kinetic parameters for [** ^**15**^ **O]H**_**2**_ **O and [** ^**18**^ **F]fluciclatide at the VOI level**

Patient number	***K***_1_[^15^O]H_2_O	***k***_2_[^15^O]H_2_O	***k***_3_/***k***_4_[^18^F]fluciclatide	SUVfluci DELIVERY	SUVfluci RETENTION
	(mL/cm^3^/min)	(L/min)	(unitless)	(m^2^/L)	(m^2^/L)
1	0.15	0.32	4.34	0.070	0.049
2	0.16	0.38	4.37	0.137	0.072
3	0.38	0.61	4.87	0.045	0.064
4	N/A	N/A	7.81	0.050	0.087
5	N/A	N/A	6.57	0.040	0.070
6	0.68	0.88	N/A	N/A	N/A
(Tumor#1)					
6	0.39	0.62	N/A	N/A	N/A
(Tumor#2)					
7	0.44	0.72	4.38	0.077	0.062

**Figure 3:**
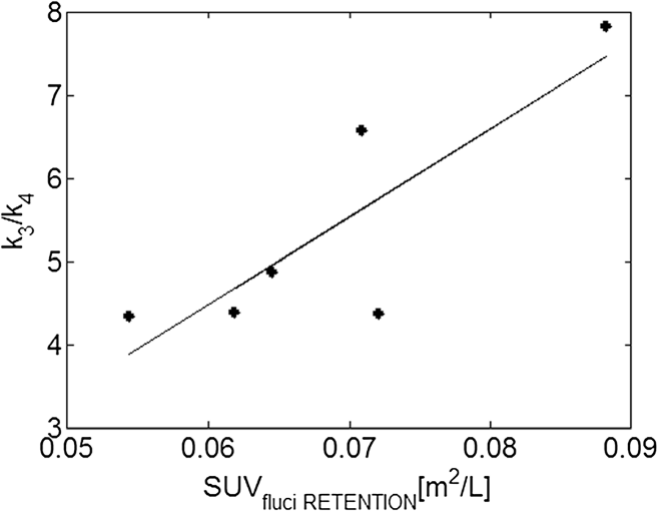
**Correlation between [** ^**18**^ **F]fluciclatide retention SUV and** ***k***_**3**_ **/** ***k***_**4**_ **ratio.**

The *k*_3_/*k*_4_ ratio of [^18^F]fluciclatide was 5.39 ± 1.46, in good agreement with our previous results in lung metastases (*k*_3_/*k*_4_ = 6.09 ± 3.04). There was a good correlation (Pearson *r* = 0.71, *p* value = 0.06, Figure 3) between SUVfluci RETENTION (*x*-axis) and *k*_3_/*k*_4_ (*y*-axis) at the VOI level supporting the use of the semi-quantitative parameter SUVfluci RETENTION as a measure of retention.

**Figure 4:**
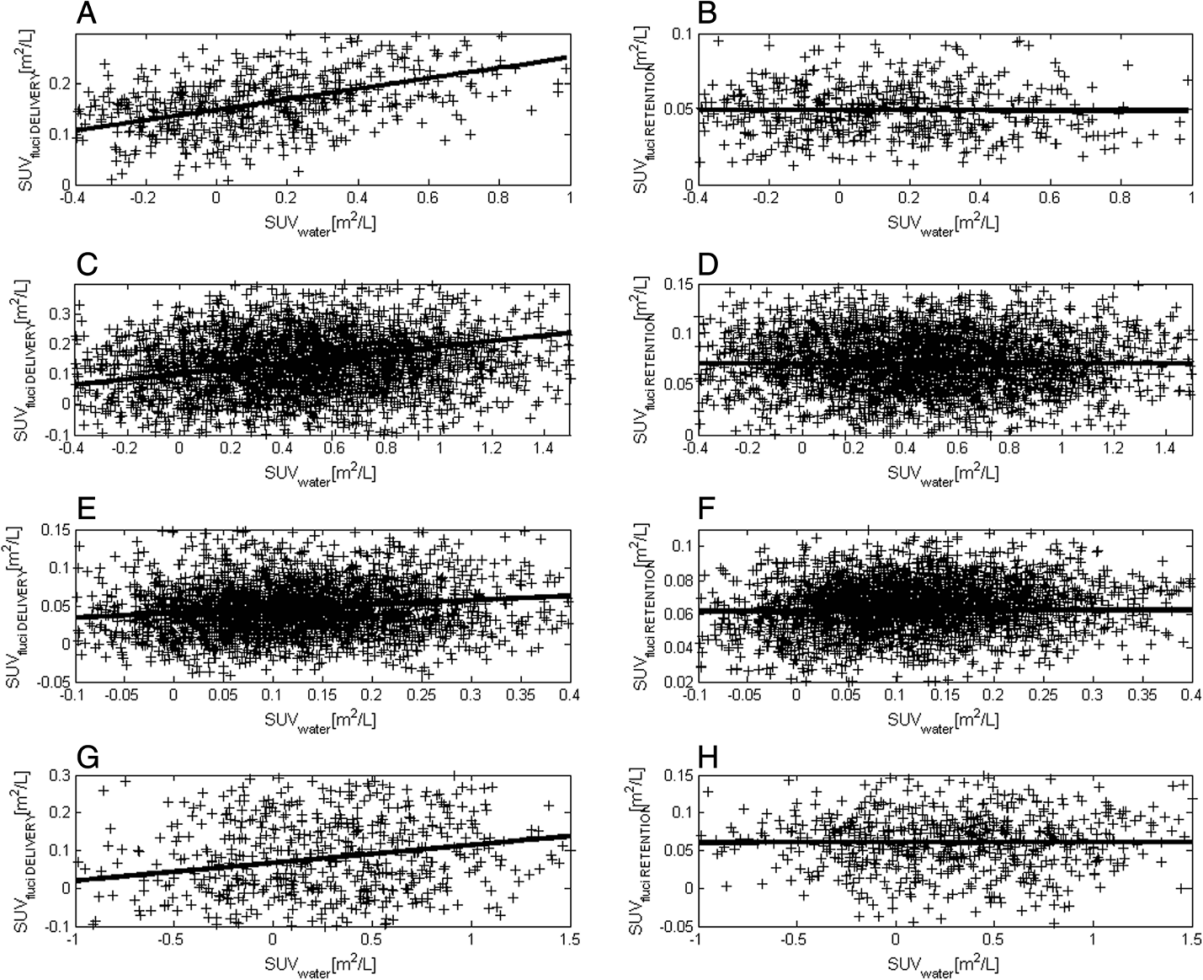
**Relationship between fluciclatide delivery and water SUV (A,C,E,G) and between SUV fluciclatide retention and SUV for water (B,D,F,H).** There was a lack of relationship between between SUV fluciclatide retention and SUV for water.

To investigate the relationship between perfusion and [^18^F]fluciclatide retention, which was not feasible at the VOI level due to the low number of subjects completing both scans, we compared SUVwater with SUVfluci RETENTION or SUVfluci DELIVERY at the voxel level. As expected, SUVwater was well correlated with SUVfluci DELIVERY, the correlation being statistically significant for voxels within all four tumors (*p* value <10^−4^ for all VOIs). The correlation between SUVwater and SUVfluci RETENTION, on the other hand, was not statistically significant (Spearman *p* value >0.15 for all tumors). This information is displayed in Figure 4, which shows the relationship between SUVwater (*x*-axis) and SUVfluci DELIVERY (*y*-axis, Figure 4A,C,E,G for patients 1, 2, 3, and 7, respectively) and between SUVwater and SUVfluci RETENTION (*y*-axis, Figure 4B,D,F,H). The plots on the same row correspond to the same patients and the continuous line is the fitted line of equation *y* = *mx* + *q*. Whereas a monotonic relationship can be noticed between SUVwater and SUVfluci DELIVERY, there is no clear visual relationship between SUVwater and SUVfluci RETENTION .

## Discussion

There is an increasing need for effective biomarkers which can reliably predict the response to novel therapeutics acting as angiogenesis inhibitors or as vascular disrupting agents *in vivo*, and the RGD-based ligand, [^18^F]fluciclatide, is very promising in this respect. Given the haemodynamic context of angiogenesis, the uptake characteristics of a good biomarker should be independent of blood flow. Consequently, the objective of this study, which is of scientific relevance to the use of this and possibly other RGD-based ligands, was to assess the dependency of [^18^F]fluciclatide uptake on perfusion. The major finding of the study is that, at the voxel level, [^18^F]fluciclatide retention is not correlated with perfusion measured by [^15^O]H_2_O uptake. We contrast this finding with a good agreement, as expected, between [^15^O]H_2_O uptake and [^18^F]fluciclatide delivery suggesting that the lack of correlation between [^15^O]H_2_O uptake and [^18^F]fluciclatide retention was not due to noise. Incidentally, the two-tissue compartment model which we validated for [^18^F]fluciclatide in our previous report proved to be appropriate also for this dataset, and the mean *k*_3_/*k*_4_ ratio obtained in this study (5.39 ± 1.46) was in good agreement with the results obtained in lung metastases (*k*_3_/*k*_4_ = 6.09 ± 3.04).

The main limit in this initial study is the small sample size, as only four out of seven patients successfully completed both scans, which hampered the quantification of the relationship between *K*_1_ of [^15^O]H_2_O and [^18^F]fluciclatide *k*_3_/*k*_4_ at the VOI level. In this regard our future plan is to test further these preliminary findings on a larger cohort of subjects and also to apply/develop appropriate noise-reduction approaches which will allow us to compare *K*_1_ maps of [^15^O]H_2_O and *k*_3_/*k*_4_ of [^18^F]fluciclatide at the voxel level.

## Conclusion

In conclusion, these preliminary results support the view that [^18^F]fluciclatide binding (retention) is not dependent on perfusion, highlighting the potential of this radiotracer in the study of receptors expressed on the neovasculature. The study supports the use of late imaging protocols (60 min post injection) for assessment of [^18^F]fluciclatide uptake.

## Authors’ original submitted files for images

Below are the links to the authors’ original submitted files for images. Authors’ original file for figure 1 Authors’ original file for figure 2 Authors’ original file for figure 3 Authors’ original file for figure 4

## Abbreviations

BSA: Body surface area
HPLC: High-performance liquid chromatography
NSCLC: Non-small cell lung cancer
RGD: Arginine-glycine-aspartame
SUV: Standardised uptake value
VOI: Volume of interest
WNLSS: Weighted non-linear least squares
WRSS: Weighted residual sum of squares
