# Supplementary figures and images for: Preliminary clinical assessment of the relationship between tumor alphavbeta3 integrin and perfusion in patients studied with [18F]fluciclatide kinetics and [15O]H2O PET
Source: EJNMMI Res. 2014 Aug 8;4:30. doi: 10.1186/s13550-014-0030-x (PMC4884000; doi:10.1186/s13550-014-0030-x)

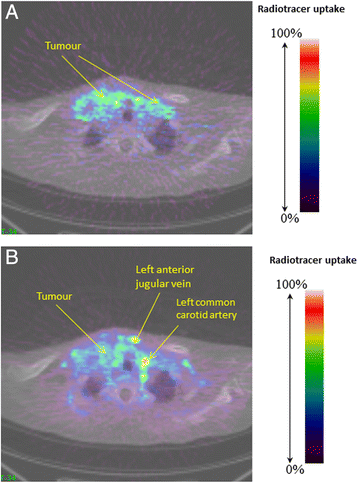

Supplement: Supplementary file 1 — Authors’ original file for figure 1 [file 13550_2014_30_MOESM1_ESM.gif]

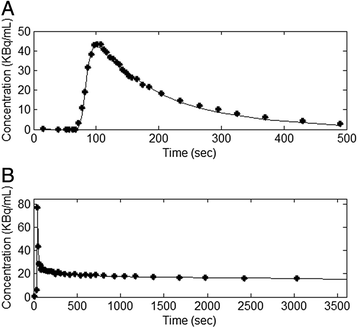

Supplement: Supplementary file 2 — Authors’ original file for figure 2 [file 13550_2014_30_MOESM2_ESM.gif]

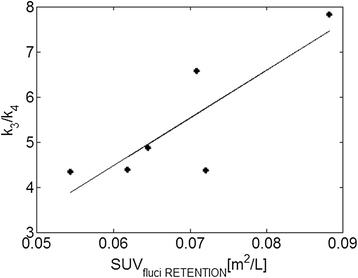

Supplement: Supplementary file 3 — Authors’ original file for figure 3 [file 13550_2014_30_MOESM3_ESM.gif]

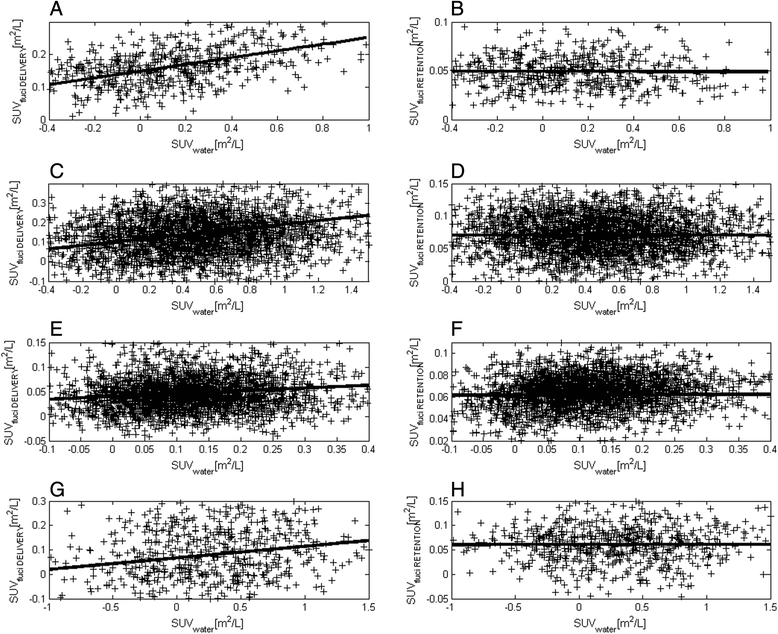

Supplement: Supplementary file 4 — Authors’ original file for figure 4 [file 13550_2014_30_MOESM4_ESM.gif]
